# Prevalence and Antimicrobial Susceptibility Profile of *Salmonella* from Poultry Farms and In-Contact Humans and Associated Risk Factors in Addis Ababa, Ethiopia

**DOI:** 10.1155/2024/4227460

**Published:** 2024-04-26

**Authors:** Aberaw Akalu, Tekalign Tadesse, Haile Alemayehu, Girmay Medhin, Desalegn Woldeyohannes, Tadesse Eguale

**Affiliations:** ^1^Food, Medicine and Healthcare Administration and Control, Addis Ababa, Ethiopia; ^2^Department of Veterinary Science, Mattu University, Mattu, Ethiopia; ^3^Aklilu Lemma Institute of Pathobiology, Addis Ababa University, Addis Ababa, Ethiopia; ^4^Department of Immunology and Molecular Microbiology, Texas Tech University Health Sciences Center, Lubbock 79430, Texas, USA; ^5^Ohio State Global One Health LLC, Addis Ababa, Ethiopia

## Abstract

Poultry and poultry products are the common sources of *Salmonella,*which is one of the serious food-borne bacterial diseases in humans. Little is known about the status of *Salmonella* and their antimicrobial susceptibility in poultry farms in Addis Ababa. This study was conducted to estimate the prevalence and antimicrobial susceptibility of *Salmonella* isolates and to investigate possible risk factors for the occurrence of *Salmonella* in poultry farms in Addis Ababa. We recruited 58 poultry farms, from which 471 poultry-related samples and 44 stool samples from in-contact humans were collected. The isolates were tested for their susceptibility to 11 antimicrobials using the Kirby–Bauer disk diffusion assay. The farm-level prevalence of *Salmonella* was 36.2% and the sample-level prevalence was 6.4% for samples taken from poultry farms and 4.5% in human stool samples who have contact with poultry. On-farm waste disposal practices and chicken being purchased from different multiplication farms were significantly associated with *Salmonella* positivity of the farms (*p* < 0.05). Eleven (34.4%) *Salmonella* isolates were resistant to streptomycin, and nine (28.1%) were resistant to tetracycline. Thirteen (40.6%) *Salmonella* isolates were resistant to two or more antimicrobials tested in this study, whereas resistance to 3 or more antimicrobials was detected in seven (21.9%) isolates. In conclusion, a high prevalence of *Salmonella* and a high rate of resistance to multiple antimicrobials were detected in poultry farms in Addis Ababa. Hence, implementation of strong biosecurity measures and rational use of antimicrobials are recommended.

## 1. Introduction

*Salmonella* is among the most common food-borne pathogens worldwide and is one of the major global public health concerns [1]. Consumption of contaminated animal-derived foods and direct contact with animals are the most common ways to get infected with nontyphoidal *Salmonella*. It has been found in a wide variety of foods, with outbreaks primarily associated with animal products, including eggs, poultry, and dairy products [2, 3]. This indicates the need of risk reduction strategies throughout the food chain.

Poultry and other food animals are major reservoirs of *Salmonella*, and undercooked poultry products are the most common source of nontyphoidal *Salmonella* infection in humans. Several *Salmonella* serotypes, which infect a wide range of animals and people, are commonly isolated from poultry without presenting any clinical symptoms [4, 5]. Numerous outbreaks have been linked to the consumption of undercooked or raw eggs, and poultry meat is particularly risky for humans. It has been shown that 45.6% of the reported salmonellosis outbreaks in the European Union (EU) were associated with the consumption of eggs and egg products [6, 7].

Multiple factors are responsible for the occurrence of *Salmonella* on poultry farms. Several researchers have identified possible factors associated with the presence of *Salmonella* in poultry farms. These include farms with large flock sizes, chicken sources from different multiplication centers, farms that use floor housing systems, and rearing layer chickens. Other management factors, such as on-farm waste disposal, contamination of feed with excreta, not using disinfectants in the farms, and those farms devoid of the rodent control system, have been associated with the occurrence of *Salmonella*. In addition, previous findings indicated that not washing hands after using the toilet and not using protective gloves while handling chickens and their products have also been associated with the prevalence of *Salmonella* [2, 5, 8–11].

In recent studies, most *Salmonella* isolates from poultry products and poultry farms have been shown to be resistant to several antimicrobials. The extent of the public health risk associated with poultry products could be described using data on farm prevalence and the antibiotic susceptibility status of isolates [5]. Studies and unpublished findings from different health institutions in Ethiopia have shown that salmonellosis is a widespread issue and that several serotypes are found in humans, poultry, animal food items, and other foods [12]. According to Eguale [5], 14.6% of the pooled fecal droppings of birds were positive for *Salmonella* in poultry farms in Central Ethiopia. A study in Southern Ethiopia showed that *Salmonella* was isolated in samples from all three farms studied at a rate of 16.7% [10]. Another study showed the prevalence of *Salmonella* in poultry farms at a rate of 15% and an 8% prevalence in eggs from the retail market [13].

Despite the rise in the number of poultry farms in urban and peri-urban Addis Ababa, there is a dearth of information on the current prevalence of *Salmonella* in poultry farms and in-contact humans in the area. Studying the rate of *Salmonella* occurrence and their antimicrobial susceptibility in urban and peri-urban areas of Addis Ababa where animals and humans live in close proximity is important to inform policy-makers and health practitioners on possible risks associated with poultry-associated salmonellosis. Hence, this study aimed to estimate the prevalence of *Salmonella,* test the antimicrobial susceptibility of *Salmonella* isolates in poultry and in-contact humans, and investigate factors associated with *Salmonella* positivity in poultry farms in Addis Ababa.

## 2. Materials and Methods

### 2.1. Study Area

**Figure 1:**
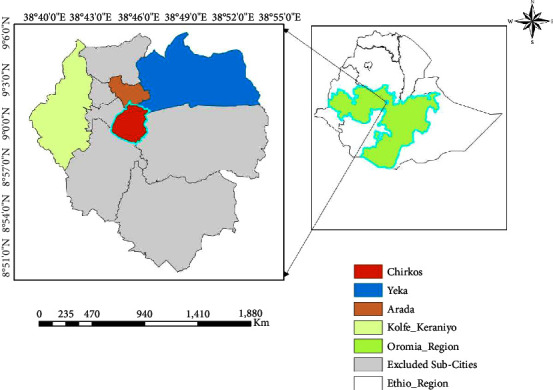
Map of the study area.

This study was conducted in Addis Ababa, located at an altitude of 2,408 meters above sea level [14] (NMSA). Based on the United Nations World Population Prospect Metro Area Population Census results, the metro area population of Addis Ababa in 2020 is 4,794,000, [15]. The city has a certain rural population, which is in the 5 expansion cities, but the 5 inner subcities are entirely urban. Addis Ababa contains 22.9% of all urban dwellers in Ethiopia (Figure 1).

### 2.2. Study Design and Study Population

A cross-sectional study was carried out on poultry farms in four subcities of Addis Ababa from November 2022 to July 2023. All poultry and people who had contact with poultry on the poultry farms in Addis Ababa were the source population for this study. The study population comprises all poultry-related materials such as fresh pooled fecal droppings, feed, and floor swabs from selected poultry farms. In addition, volunteer farm attendants who have direct contact with poultry were included in the study population. Poultry farms with a flock size of a minimum of 100 birds, farm owners, and in-contact humans who volunteered to participate in the study were included.

### 2.3. Sample Size Determination

The minimum required sample size was calculated based on previous work that reported a 16.7% sample-level prevalence of *Salmonella* in Southern Ethiopia [10] with a 95% desired confidence interval and 5% absolute precision using the formula described previously [16]. Using 2 design effects as a correction factor and a 10% nonresponse rate, the total calculated sample size was 471 [17]. The number of pooled fecal droppings collected from each farm was based on the farm size. In the case of larger-sized farms, the required number of fresh fecal droppings was proportionally increased. Besides, to assess the contamination of poultry environments with *Salmonella*, feed and floor swab samples were also collected without impairing the representativeness of the sample.

#### 2.3.1. Sampling Techniques

A multistage sampling method was employed. Accordingly, all subcities in Addis Ababa were divided into two categories based on their geographical location (inner subcities and expansion subcities) and the number of poultry farms. From each category, two subcities and a total of four subcities were selected for this study. Based on this, Arada and Kirkos were selected from the inner subcities, and Yeka and Kolfe Keranio subcities were selected from the expansion subcities randomly. Subsequently, Woredas (smallest administrative units) were selected from the selected subcities for the study, from which representative, poultry farms were selected.

The list of poultry farms with flock sizes for all Woredas was obtained from the Addis Ababa City Administration Agriculture Office. A total of 12 Woredas were then selected from both expansion subcities and inner subcities according to the number of Woredas found in each subcity. Accordingly, 8 Woredas from expansion subcities and 4 Woredas from inner subcities were selected randomly. Of the total 112 poultry farms found in the selected 12 Woredas, 58 poultry farms were selected for this study according to the number of poultry farms found per Woreda. In this study, farms containing less than 500 birds were considered small poultry farms, whereas those containing more than 500 birds were considered large poultry farms [2].

### 2.4. Sample and Data Collection and Transportation

A total of 471 poultry-related samples were collected from different farms after obtaining verbal consent from the respondents. These samples comprise 330 pooled fresh fecal droppings, 70 floor swabs, and 71 feed samples. In addition, 44 stool samples were collected from consented volunteer farm attendants after explaining the purpose and procedure of the study. Fresh fecal droppings weighing at least 5 gm were collected with clean disposable gloves and placed in sterile plastic bags with a zipper. For the farm containing 100–200 flock sizes, 4 pooled fecal droppings (3 pooled droppings) were collected to give a good representation of the farm [5].

In the same way, a minimum of 5 gm of poultry feed sample was collected from each poultry farm in a sterile container. Floor swabs were collected from each poultry farm by swabbing a 10 cm by 10 cm area of the floor where the birds were kept with a buffered peptone water (BPW)-moistened sterile cotton swab. Two swab samples from 2 different corners were pooled within 10 ml of BPW. In addition, a minimum of 1 gm of stool sample was collected from farm attendants in a clean stool cup with an applicator stick [2].

In addition to poultry-related and stool samples for *Salmonella* isolation, information such as general farm management, sociodemographic characteristics of the farm attendants, and other possible risk factors was collected by interviewing the farm owners during sample collection using a standard questionnaire. The questionnaire was prepared in English and translated into Amharic for the interview, which was translated back to English. Each sample was coded, packaged separately in an icebox, and transported to the Microbiology Laboratory of Aklilu Lemma Institute of Pathobiology, Addis Ababa University, within 3–4 hrs of collection.

### 2.5. *Salmonella* Isolation and Identification

*Salmonella* isolation and identification were carried out according to the Global Foodborne Infections Network Laboratory Protocol [18]. The bacteriological media used for the study were prepared following the instructions of the manufacturers. Briefly, for isolation and identification of *Salmonella*, 5 gm of fecal droppings from birds and 1 gm of stool sample from in-contact humans were pre-enriched in 45 ml and 9 ml of sterile buffered peptone water (BPW), respectively (Oxoid), and incubated for 24 hours at 37°C. Swab samples placed in BPW were also incubated at 37°C for 24 hours. Similarly, 5 gm of feed sample was pre-enriched in 45 ml of BPW and incubated at a similar temperature overnight. From samples incubated in the primary nonselective enrichment media, 0.1 ml of the suspension was inoculated into the Modified Rappaport Vassiliadis (Oxoid, Basingstoke, England) broth media and incubated at 42°C for 24 hours. At the same time, 1 ml of the suspension was also inoculated into tetrathionate broth (Oxoid, Basingstoke, England) media and incubated at 37°C for 24 hours.

A loopful of suspension from selective enrichment media was inoculated on xylose lysine deoxycholate (XLD) agar (Oxoid, Basingstoke, England) plates. The plates were then incubated aerobically at 37°C for 24–48 hours. Typical presumptive small red translucent *Salmonella* colonies on XLD agar with a central black spot were detected. The suspected colonies were streaked on the surface of nutrient agar (Oxoid, Basingstoke, England) plates and incubated at 37°C for 24 hours for further confirmation by biochemical tests and PCR.

The biochemical tests for suspected *Salmonella* colonies were performed using triple sugar iron (TSI) agar, lysine iron agar (LIA), urease, citrate utilization test, and motility test as described previously [19]. Isolates showing specific biochemical characteristics of *Salmonella* were further confirmed using *Salmonella* genus-specific PCR as previously described [20]. The reference strain of *Salmonella enterica* serovar Typhimurium ATCC 13311 was used as a positive control during isolation and conduction of PCR. The PCR-confirmed *Salmonella* isolate from each positive sample was preserved at −80°C in 20% glycerol until further testing.

### 2.6. Antimicrobial Susceptibility Testing

The antimicrobial susceptibility test was performed according to the Clinical Laboratory Standards Institute (CLSI) guideline using the Kirby–Bauer disk diffusion method on Muller–Hinton agar plates (Oxoid, Basingstoke, England). Antimicrobials used in this study were ampicillin (10 *μ*g), nalidixic acid (30 *µ*g), sulfamethoxazole + trimethoprim (23.75 *μ*g), chloramphenicol (30 *μ*g), cephalothin (30 *μ*g), amoxicillin + clavulanic acid (20/10 *µ*g), streptomycin (10 *μ*g), ciprofloxacin (5 *μ*g), tetracycline (30 *μ*g), gentamicin (10 *μ*g), and amikacin (30 *μ*g). Antimicrobial disks used in this study were all obtained from Sensi-Disc, Becton, Dickinson and Company. *Escherichia coli* ATCC 25922 strains were used as a control during the antimicrobial susceptibility test. *Salmonella* isolates were considered multidrug-resistant when they were resistant to two or more antimicrobials belonging to different classes. The interpretation of the susceptibility test result is based on the CLSI guideline [21].

### 2.7. Data Analysis

Data were collected using Open Data Kit (ODK) version 1.30.1 and exported to SPSS Windows version 26.0 (SPSS Inc., Chicago, IL) for statistical analysis. Frequency tables and descriptive summaries were used to describe the study variables. The sample-level prevalence of *Salmonella* was calculated as a percentage of *Salmonella* culture-positive samples, whereas the farm-level prevalence was calculated as the percentage of farms with one or more *Salmonella* culture-positive samples among the total farms sampled. A chi-square test was used to assess the association between *Salmonella* occurrence and explanatory variables at the farm level, while an adjusted odds ratio (AOR) at a 95% confidence interval was used to measure the association between potential risk factors and the occurrence of *Salmonella* at the sample level. The model fit was assessed using the Hosmer–Lemeshow test (goodness of fit), and it had an acceptable fit to the data (*p* > 0.05). All independent variables with *p* < 0.2 in the univariable logistic regression were included in the multivariable logistic regression. In the multivariable logistic regression, independent variables with a *p* < 0.05 were considered as having a significant association with the outcome of interest.

### 2.8. Ethical Consideration

\The study was approved by the Institutional Review Board of Aklilu Lemma Institute of Pathobiology, Addis Ababa University (Ref. No. *ALIPB/IRB/37/2013/21*). A formal letter was written to poultry farms to get permission and cooperation to conduct the study. Poultry farm owners were requested to participate in the study, and individual informed verbal consent was obtained from poultry in-contact human subjects willing to participate in the study. They were informed that participation is solely based on their willingness. Any information obtained from participants during the study was kept confidential.

## 3. Results

### 3.1. Characteristics of Poultry Farms in the Study Area

Thirty four poultry farms (58.6%) had a small flock size from which 172 (36.5%) samples were collected for this study. Thirty-three (56.9%) farms dispose of the waste from farms outside of the farm environment. The sources of poultry for thirty seven (63.8%) farms were a single multiplication center, while the remaining farms had chickens that came from different sources. Fifty one poultry farms (87.9%) were established for egg production purposes, and there was no history of antimicrobial use in thirty eight (65.5%) farms during the last 6 months. Disinfectants were used only in thirty eight (65.5%) farms. The poultry feed was observed to be contaminated with chicken excreta in twelve (20.7) poultry farms (Table 1).

### 3.2. Prevalence of *Salmonella* in Poultry Farms and In-Contact Humans

**Figure 2:**
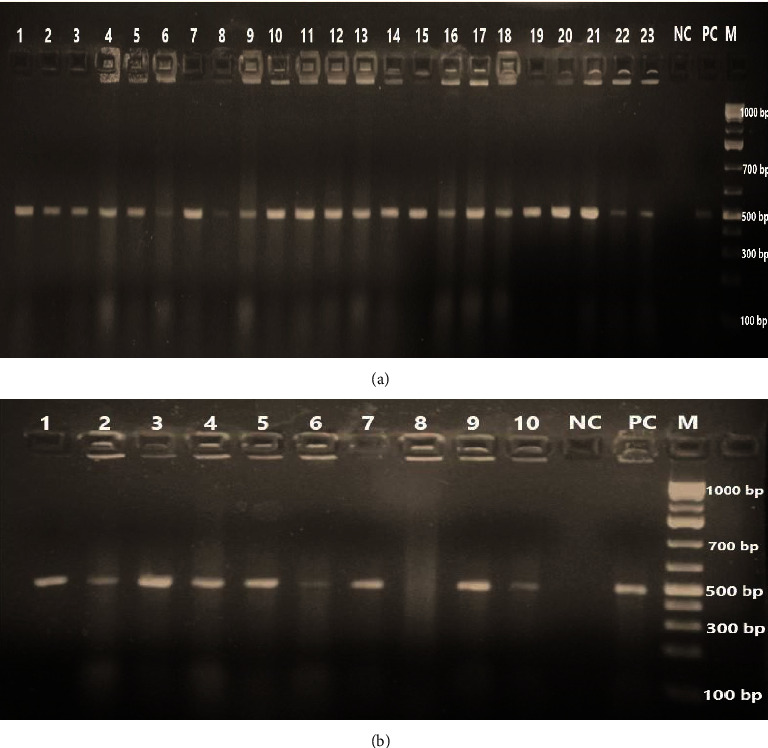
PCR gel image of 496 bp amplification of Salmonella genus-specific PCR when run on 2% agarose gel. (a) Lanes: M, 100 bp DNA ladder; 1-23 positive for Salmonella (496 bp), PC-positive control, and NC for negative control. (b) Lanes: M, 100 bp DNA ladder; 1-7, 9, and 10 positives for Salmonella (496 bp), PC-positive control while NC - negative control.

A gel image of the *Salmonella* genus-specific PCR is shown in Figure 2.

*Salmonella* was isolated from 36.2% (21/58) of the studied farms and 6.4% (30/471) of different poultry-related samples collected in the current study, and 4.5% (2/44) of the human stool samples were positive for *Salmonella*. *Salmonella* isolation was more common in farms from the Kirkos subcity as compared to the other subcities; however, this difference was not statistically significant (Table 2).

### 3.3. Factors Associated with *Salmonella* Occurrence in Poultry Farms

Twenty-one (36.2%) of the 58 poultry farms were contaminated with *Salmonella*. Isolation was significantly more common in large flock-sized farms (*p*=0.005). The occurrence of *Salmonella* was significantly higher in chickens established for egg production, farms not using disinfectants (*p* < 0.001), and those practicing on-farm waste disposal (*p*=0.002). Similarly, *Salmonella* isolation was also significantly higher in farms where poultry feed was found to be contaminated with chicken excreta (*p*=0.02), and the source of birds was from different poultry multiplication centers (*p*=0.022) (Table 3).

The level of *Salmonella* isolation varied depending on the type of sample: 22 (6.7%) pooled fecal droppings, 5 (7.14%) floor swabs, and 3 (4.3%) feed samples were positive for *Salmonella*. The isolation of *Salmonella* in different poultry-related samples was significantly associated with the observation of chicken feed contaminated with chicken excreta (*χ*^2^ = 0.175, *p*=0.008) and on-farm waste disposal practice (*χ*^2^ = 6.313, *p*=0.013). Farms that received chicken from different multiplication centers and those not using disinfectants were significantly associated with the presence of *Salmonella* (*p* < 0.05). *Salmonella* was detected in only two of 44 stool samples of people who had contact with poultry farms (Table 4).

On-farm waste disposal practices, receiving chicken from different multiplication centers, farms that do not use disinfectants, and contamination of feed with chicken excreta were significantly associated with the detection of *Salmonella* in various poultry-related samples. These variables were considered for multivariable regression modeling. Findings from the multivariable logistic model identified are summarized in Table 5. On-farm waste disposal practice was associated with an increased odds ratio of the birds being positive for *Salmonella* (adjusted odds ratio (AOR) = 3.184; 95% CI: 1.251–8.107), while observation of poultry feed not contaminated with chicken excreta was associated with a lower odds of being positive for *Salmonella* (AOR = 0.300; 95% CI: 0.119–0.753). The odds of *Salmonella* positivity was significantly higher in farms that receive chickens from different multiplication centers (AOR = 3.146; 95% CI: 1.331–7.438) than in farms that receive chickens from a single multiplication center.

### 3.4. Antimicrobials Used in Poultry Farms

Only 34.5% of the study farms (20/58) reported the use of antimicrobials in the last 6 months. Oxytetracycline was used in 8 farms (40%) that use antimicrobials, and streptomycin was used in the feed or water in 6 (30%) of 20 farms. The other antimicrobials were amoxicillin + clavulanic acid 15%; 3/20, sulfamethoxazole + trimethoprim 10%; 2/20, and ampicillin 5%; 1/20. In addition to antimicrobials intended for poultry farming, 25% (5/20) of the farms that use antimicrobials also reported the use of tetracycline tablets, which were intended for human use. Of the 21 *Salmonella*-positive farms, 11 (55%) were from poultry farms that used antimicrobials, while 10 (26.3%) were from poultry farms that did not use antimicrobials. All the farms use antimicrobials for therapeutic and prophylactic purposes when one or more sick birds are present in the flock. However, antimicrobials were not used as a feed additive on any of the visited farms.

Of the 38 poultry farms that reported the use of disinfectants as foot baths, sodium hypochlorite 20 (52.6%), hydrogen peroxide 113 (34.2%), and formalin 5 (13.2%) were used for cleaning poultry houses before the introduction of new stock and for cleaning feeding utensils. Similarly, 5 (8.6%) total poultry farms reported that zinc phosphate was used as a rodenticide. The remaining 20 (34.5%) and 53 (91.4%) poultry farms were not using any disinfectants or rodenticides, respectively (Table 6).

### 3.5. Antimicrobial Susceptibility of *Salmonella* Isolates from Poultry Farms

**Figure 3:**
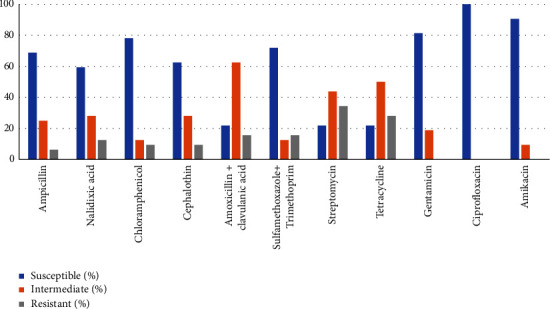
Antimicrobial susceptibility profile of *Salmonella* isolates from poultry farms and in-contact humans in Addis Ababa.

The antimicrobial susceptibility of *Salmonella* isolates investigated in the current study is presented in Figure 3. All isolates were completely susceptible to ciprofloxacin, gentamicin, and amikacin. However, intermediate or complete resistance to other antimicrobials was detected among the isolates. Resistance to streptomycin, tetracycline, amoxicillin + clavulanic acid, sulfamethoxazole + trimethoprim, and nalidixic acid was observed at the rates of 34% (11/32), 28.1% (9/32), 15.6% (5/32), 15.6% (5/32), and 12.5% (4/32), respectively (Figure 3).

Overall, 11 different resistance patterns were detected among *Salmonella* isolates investigated in this study. Nineteen (59.4%) of the 32 *Salmonella* isolates did not show complete resistance to any of the antimicrobials tested. Resistance to 2 or more antimicrobials was detected in 13 (40.6%) isolates, whereas resistance to 3 or more antimicrobials was detected in 7 (21.9%) isolates. Resistance to two or more antimicrobials was detected more commonly on farms using antimicrobials. Two (6.3%) isolates were resistant to 6 antimicrobials tested with a common resistance pattern of NA-C-AMC-SXT-S-Te. Of the 2 *Salmonella* strains from humans, the resistance pattern of one *Salmonella* isolate was similar to the *Salmonella* isolate obtained from the fresh pooled fecal sample of chickens on the farm where the individual worked. Both *Salmonella* isolates from humans and pooled fecal samples were resistant to streptomycin, tetracycline, nalidixic acid, chloramphenicol, amoxicillin + clavulanic acid, and sulfamethoxazole + trimethoprim (Table 7).

## 4. Discussion

The observed prevalence of *Salmonella* in poultry farms without detectable clinical signs, followed by exposure of in-contact humans to contaminated waste and poultry products, could be a source of human salmonellosis. Although the use of antimicrobials in poultry farms has a crucial role in the treatment and control of *Salmonella* and other bacterial pathogens, unnecessary overuse and misuse of antimicrobials could lead to the emergence and spread of multidrug resistance. In the current study, the farm-level prevalence of *Salmonella* in poultry farms was 36.2%, which is higher than other studies conducted in Adama and Modjo, Ethiopia (28.8%) [2], and Central Ethiopia (14.6%) [5]. However, it is much lower than a report from Nigeria [8] and Nepal [22] of 47.9% and 55%, respectively.

The sample-level prevalence of *Salmonella* was 6.4% in the current study, which is higher than similar studies in Adama and Modjo towns (2.8%), Central Ethiopia (4.7%), and in and around Modjo and Central Oromia [2, 5, 23]. However, it is lower than the 19% prevalence reported from West Showa, Ethiopia [24] (Sarba et al.). Such variation could be due to factors such as differences in detection methods employed, farm biosecurity level, and hygienic practices in the farms. Most of the farms in the current study were in the same compound with human residential areas or close to each other, and the chance of transmitting *Salmonella* from one farm to other and from humans to chickens and vice versa is high. The possible reason for the low prevalence compared with reports in other countries could be due to the smaller size of most of the farms in the current study. Previous studies indicated that the larger the farm size, the higher the chance of being contaminated with *Salmonella* [2].

The occurrence of *Salmonella* was significantly higher in large flock-sized farms than in small flock-sized farms, which is in line with the findings reported in Central Ethiopia [5], Adama and Modjo [2], and elsewhere [8, 25]. The possible explanation is that once large flocks are infected, the infection spreads quickly due to overcrowding, and the possibility of persisting in the farm is high compared with small flock-sized farms. The reason for the higher rate of *Salmonella* in layer flocks than broiler flocks could be that layer flocks spend more time in the poultry house and exhibit a greater chance of being infected with *Salmonella*. Similarly, other workers reported a significantly higher rate of *Salmonella* detection in layer flocks than in broilers in Sweden [26] and Nigeria [8].

Interestingly, unlike other previous reports from Ethiopia [5, 9], a high rate of *Salmonella* was detected in farms that did not use disinfectants compared with farms that used disinfectants in the current study. The possible reason might be linked to the introduction of *Salmonella* into the poultry farms via uncleaned fomites and people entering the farms if there is no disinfectant containing footbath. This agrees with the previous report by [8] from Nigeria.

The detection of *Salmonella* was significantly higher in farms that practice on-farm waste disposal than in those that practice off-farm waste disposal. The reason for this could be that on-farm disposed wastes might be a source of contamination for the chickens on the farm, and there is the potential for on-farm transmission of *Salmonella*. This finding completely agrees with previous reports from African countries [8, 10, 27].

Farms, where poultry feed was found to be contaminated with chicken excreta, had a higher rate of *Salmonella* detection than those where poultry feed was not contaminated with chicken excreta. The possible reason could be that chickens most likely share the excreta-contaminated feeds, thereby increasing the chance of the spread of *Salmonella*. This finding is closely in line with the studies of South-Western Ethiopia [9].

In addition, a significant association was observed between *Salmonella* prevalence and farms that received chickens from different multiplication centers compared with farms that received chickens from a single multiplication center. The possible explanation for this could be due to the increased chance of introducing S*almonella* from different contaminated farms. This finding agrees with previous reports from South Ethiopia [10] and a study from Uganda [27].

The prevalence of *Salmonella* among in-contact humans showed a slightly higher rate than previous studies in Adama and Modjo [2, 28], but the rate is still low. The main reason for this low rate could be due to the small sample size. Second, most poultry farm attendants wash their hands with soap or disinfectants after having contact with poultry. The majority of the in-contact study participants responded that they handle poultry and poultry products using protective gloves. As a result, the risk of being exposed to *Salmonella* might have been low because of these and other personal hygienic practices.

In this study, resistance was detected more frequently to antimicrobials reported to be used in farms such as streptomycin, tetracycline, amoxicillin + clavulanic acid, and sulfamethoxazole + trimethoprim. The highest rate of resistance was detected for streptomycin. This antimicrobial has long been used in Ethiopia in veterinary medicine together with penicillin [29]. The high rate of resistance to streptomycin and tetracycline in the current study is in agreement with previous reports in Ethiopia [2, 5, 10] and elsewhere [4]. The possible reason for such a high rate of resistance could be that chicken and other animal owners in Addis Ababa commonly use these antimicrobials without prescription simply, resulting in an increased rate of selection for resistant isolates. Furthermore, once resistant isolates started to occur in a farm, frequent use of antimicrobials in the farm and experts' selection pressure on other susceptible bacterial species lead to the increased chance of multiplication of resistant *Salmonella* isolates, thereby increasing the rate of detection of resistant isolates. This agrees with the previous report from Ethiopia [2, 5, 10].

The presence of isolates resistant to two or more antimicrobials in this study agrees with previous findings in the USA and previous studies in Ethiopia [10, 30]. The detection of isolates resistant to 6 antimicrobials in two of the isolates is worrying. The fact that these isolates were obtained from poultry in a farm and stool samples of in-contact humans working in the same farm suggests transmission of multidrug-resistant isolates between poultry and humans.

## 5. Conclusion

The current study showed a high prevalence of *Salmonella* on poultry farms, demonstrating a high public health risk associated with *Salmonella* originating from poultry in the study area. Farms with large flock sizes, on-farm poultry waste disposal practices, receiving chicken from different multiplication centers, not using disinfectants in the farm, layers compared with broilers, and contamination of poultry feed with chicken excreta were significantly associated with farm-level *Salmonella* positivity. Resistance to streptomycin and tetracycline was detected in a substantial proportion of *Salmonella* isolates. Standard poultry husbandry practices such as proper disposal of poultry waste material, avoiding contamination of poultry feed with the excreta of chickens, regular disinfection of farms, and application of farm biosecurity should be applied to minimize contamination of poultry with *Salmonella*. In addition, awareness creation on the prudent use of antimicrobials by poultry farmers in the study area is recommended to minimize the burden of antimicrobial resistance.

## Figures and Tables

**Table 1:** Distribution of poultry farms involved in the current study across subcities and their characteristics.

Study variables	No. (%) of farms	No. (%) of samples
	(*n* = 58)	(*n* = 471)
*Studied subcities*		
Arada	9 (15.5)	89 (18.9)
Kirkos	5 (8.6)	33 (7.0)
Yeka	24 (41.4)	197 (41.8)
Kolfe Keranio	20 (34.5)	152 (32.3)

*Flock size of chickens*		
Large	24 (41.4)	299 (63.5)
Small	34 (58.6)	172 (36.5)

*Age of chickens*		
<6 months	28 (48.3)	231 (49.0)
6–12 months	26 (44.8)	219 (46.5)
>12 months	4 (6.9)	21 (4.5)

*Poultry farm's waste disposal practices*		
On-farm waste disposal	25 (43.1)	257 (54.6)
Off-farm waste disposal	33 (56.9)	214 (45.4)

*Source of birds*		
Received from different multiplication centers	21 (36.2)	180 (38.2)
Received from a single multiplication center	37 (63.8)	291 (61.8)

*Use of disinfectants*		
No	20 (34.5)	171 (36.3)
Yes	38 (65.5)	300 (63.7)

*Antimicrobial use history during the last 6 months*		
No	38 (65.5)	314 (66.7)
Yes	20 (34.5)	157 (33.3)

*Type of commodity*		
Layers	51 (87.9)	431 (91.5)
Broilers	7 (12.1)	40 (8.5)

*Type of chicken-keeping house*		
Floor system	26 (44.8)	232 (49.3)
Cage system	32 (55.2)	239 (50.7)

*Is poultry feed contaminated with chicken excreta?*		
No	46 (79.3)	354 (75.2)
Yes	12 (20.7)	117 (24.8)

**Table 2:** Prevalence of *Salmonella* in poultry farms and in-contact humans in Addis Ababa.

Studied subcities	No. of tested			No. (%) of positive		
	Farms (*n* = 58)	Poultry-related samples (*n* = 471)	Stool samples (*n* = 44)	Farms	Poultry-related samples	Stool samples
Arada	9	89	6	4 (44.4)	9 (10.1)	0 (0.0)
Kirkos	5	33	4	3 (60.0)	4 (12.1)	1 (25.0)
Yeka	24	197	29	7 (29.2)	7 (3.6)	1 (3.4)
Kolfe Keranio	20	152	5	7 (35.0)	10 (6.6)	0 (0.0)
Total	58	471	44	21 (36.2)	30 (6.4)	2 (4.5)

**Table 3:** Association of farm-level factors with the presence of *Salmonella* in poultry farms in Addis Ababa.

Characteristics	Categories	No. of farms tested	No. (%) of positive farms	Chi-square	*p* value
Flock size of chickens	Large (≥500)	24	14 (58.3)	8.678	0.005
	Small (<500)	34	7 (20.6)		

Poultry farm's waste disposal practices	On-farm waste disposal	25	15 (60.0)	10.769	0.002
	Off-farm waste disposal	33	6 (18.2)		

Source of chicken	Different multiplication centers	21	12 (57.1)	6.247	0.022
	A single multiplication center	37	9 (24.3)		

Chicken housing system	Floor system	26	12 (42.9)	1.037	0.414
	Cage system	32	9 (30.0)		

Use of disinfectant	No	20	14 (70.0)	15.093	0.001
	Yes	38	7 (18.4)		

Age of chickens	<6 months	28	2 (42.9)	1.086	0.581
	6–12 months	26	8 (30.8)		
	>12 months	4	1 (25.0)		

Type of commodity	Layers	51	19 (37.3)	0.201	<0.001
	Broilers	7	2 (28.6)		

Wash hands after use of the toilet	No	12	6 (50.0)	1.246	0.320
	Yes	46	15 (32.6)		

Chicken excreta mixed with poultry feed	No	46	13 (28.3)	6.078	0.020
	Yes	12	8 (66.7)		

Presence of rodents in a farm	No	5	2 (40.0)	0.034	1.000
	Yes	53	9 (17.0)		

Use of protective gloves when handling chickens and eggs	No	15	8 (53.3)	2.569	0.129
	Yes	43	13 (30.2)		

**Table 4:** Sample-level prevalence of *Salmonella* and associated factors in poultry farms in Addis Ababa.

Characteristics	Categories	No. of samples tested	No. (%) of positive samples	Chi-square	*p* value
Flock size of chickens	Large (≥500)	299	21 (7.0)	0.587	0.558
	Small (<500)	172	9 (5.2)		

Poultry farm's waste disposal practices	On-farm waste disposal	257	23 (9.0)	6.313	0.013
	Off-farm waste disposal	214	7 (3.3)		

Source of chickens	Different multiplication centers	180	18 (10.0)	6.439	0.018
	A single multiplication center	291	12 (4.1)		

Type of chicken-keeping house	Floor system	232	19 (7.5)	1.192	0.345
	Cage system	239	11 (5.1)		

Use of disinfectant	No	171	18 (10.5)	7.779	0.010
	Yes	300	12 (4.0)		

Age of chickens	<6 months	231	17 (7.4)	0.761	0.683
	6–12 months	219	12 (5.5)		
	>12 months	21	1 (4.8)		

Type of commodity	Layers	431	28 (6.5)	0.137	1.000
	Broilers	40	2 (5.0)		

Wash hands after use of the toilet	No	113	10 (8.9)	1.533	0.267
	Yes	358	20 (5.6)		

Poultry feed contaminated with chicken excreta	No	354	16 (4.5)	0.175	0.008
	Yes	117	14 (12.0)		

Presence of rodents in a farm	No	41	2 (4.9)	0.167	1.000
	Yes	430	28 (6.5)		

Use of protective gloves when handling chickens and eggs	No	111	11 (9.9)	3.052	0.116
	Yes	360	19 (5.3)		

Types of samples	Pooled fecal droppings	330	22 (6.7)	0.666	0.717
	Floor swab samples	70	5 (7.1)		
	Feed samples	71	3 (4.2)		

**Table 5:** Summary findings from a logistic regression that investigated the association of sample-level *Salmonella* positivity with preselected factors in poultry farms.

Selected factors	Univariable		Multivariable	
	COR (95% CI)	*p* value	AOR (95% CI)	*p* value
*Poultry farm*'*s waste disposal practices*				
On-farm waste disposal	2.907 (1.222–6.913)	0.016	3.184 (1.251–8.107)	0.015

*Source of chickens*				
Different multiplication centers	2.583 (1.213–5.500)	0.014	3.146 (1.3317.438)	0.009

*Use of disinfectants*				
No	2.824 (1.325–6.015)	0.007	1.946 (0.839–4.516)	0.121

*Feed contaminated with chicken excreta*				
No	0.348 (0.164–0.738)	0.006	0.300 (0.119–0.753)	0.010

*Use of protective gloves when handling chickens and eggs*				
No	1.974 (0.909–4.286)	0.086	1.149 (0.490–2.699)	0.749

**Table 6:** Recent use of antimicrobials and the occurrence of *Salmonella* in poultry farms.

Types of antimicrobials used	No. of farms	No. (%) of *Salmonella*-positive farms
Oxytetracycline	8	5 (62.5)
Streptomycin	6	3 (50.0)
Amoxicillin + clavulanic acid	3	1 (33.3)
Sulfamethoxazole + trimethoprim	2	1 (50.0)
Ampicillin	1	1 (100)
Do not use antimicrobials	38	10 (26.3)
Total	58	21 (36.2)

**Table 7:** Resistance patterns of *Salmonella* isolated from poultry farms and in-contact humans in Addis Ababa.

S. no	R pattern	No. of antimicrobials to which isolates were resistant	No. (%) of isolates with this pattern
1	—	0	19 (59.4)
2	Amc-S	2	1 (3.1)
3	Cf-Te	2	1 (3.1)
4	S-Te	2	3 (9.4)
5	Sxt-Am	2	1 (3.1)
6	C-S-Te	3	1 (3.1)
7	Na-Amc-S	3	1 (3.1)
8	Na-Cf-S	3	1 (3.1)
9	Amc-Sxt-S-Te	4	1 (3.1)
10	Am-Cf-Sxt-S-Te	5	1 (3.1)
11	Na-C-Amc-Sxt-S-Te	6	2 (6.3)
Total			32 (100)

Am = ampicillin; Cf = cephalothin; Amc = amoxicillin + clavulanic acid; Sxt = sulfamethoxazole + trimethoprim; Na = nalidixic acid; Te = tetracycline; S = streptomycin; C = chloramphenicol.

## Data Availability

The data that support the findings of this study are made available from the corresponding author upon reasonable request.
